# Validity and reliability of the Health Literacy Assessment Scale for adherence to drug treatment among diabetics

**DOI:** 10.31744/einstein_journal/2019AO4405

**Published:** 2019-03-28

**Authors:** Maria Clara Lélis Ramos Cardoso, Aline Soares Figueiredo Santos, Adélia Dayane Guimarães Fonseca, Renê Ferreira da Silva, Priscilla Durães de Carvalho, Andrea Maria Eleutério de Barros Lima Martins

**Affiliations:** 1 Universidade Estadual de Montes Claros, Montes Claros, MG, Brazil.

**Keywords:** Health literacy, Diabetes mellitus, Medication adherence, Validation studies, Reproducibility of results, Alfabetização em saúde, Diabetes mellitus, Adesão à medicação, Estudos de validação, Reprodutibilidade dos testes

## Abstract

**Objective:**

To prepare an instrument to evaluate health literacy with regard to adherence to drug treatment among diabetics, identify the validity of its content, and estimate its reliability.

**Methods:**

Pilot study, with the following stages of instrument construction: literature review, content validation, reliability estimation (internal consistency/Cronbach’s alpha and reproducibility/Kappa).

**Results:**

The validity of content was completed and presented alpha=0.77 and Kappa values ranged from 0.31 to 1.00.

**Conclusion:**

The instrument was approved regarding content validity, presented acceptable internal consistency and reproducibility. However, when applied, measurement errors it can produce must be considered.

## RESUMO

**Objetivo:**

Elaborar um instrumento para avaliar a alfabetização em saúde relacionada à adesão medicamentosa entre diabéticos, identificar a validade de seu conteúdo e estimar sua confiabilidade.

**Métodos:**

Estudo piloto, cujas etapas de construção do instrumento foram revisão de literatura, validação de conteúdo e estimativa da confiabilidade (consistência interna/alfa de Cronbach e reprodutibilidade/Kappa).

**Resultados:**

A validade de conteúdo foi concluída e apresentou valor de alfa=0,77 e o Kappa variou de 0,31 a 1,00.

**Conclusão:**

O instrumento foi aprovado quanto à validade de conteúdo, apresentou consistência interna e reprodutibilidade aceitáveis. No entanto, ao ser utilizado, devem ser considerados os erros de medida que ele pode produzir.

## INTRODUCTION

The demographic and nutritional transitions observed in the last decades have increased morbidity and mortality from chronic non-communicable diseases (CNCD).^(^ ^1^ ^)^ Physiological and/or functional alterations during the aging process increase the risk of CNCDs. In 2011, *diabetes mellitus* (DM) stood out among CNCDs in the world,^(^ ^2^ ^)^ and in Brazil,^(^ ^1^ ^)^ and is considered a pandemic and one of the ten major causes of death. Mortality from DM decreased between 1996 and 2011, and among Brazilian adults ≤69 years old. However, the prevalence of DM is still high, especially among elderly Brazilian individuals, affecting about 20% of individuals ≥60 years old.^(^ ^3^ ^)^ In 1985, there were an estimated 30 million adults with DM worldwide. Such estimation reached 135 million in 1995, 173 million in 2002, and is expected to reach 300 million by 2030.^(^ ^4^ ^)^

*Diabetes mellitus* is a metabolic disease characterized by hyperglycemia caused by problems with insulin secretion and/or action, which also affects lipid and protein metabolism.^(^ ^5^ ^)^ The main risk factors of type 2 DM (DM2) are modifiable and include smoking, a sedentary life style, unhealthy eating habits, and excessive alcohol consumption.^(^ ^6^ ^)^ Social determinants include social inequalities, access to goods and services, low levels of education, and discrepant access to information.^(^ ^7^ ^)^ *Diabetes mellitus* requires continuous treatment, and patients need to have healthy habits, adhere to the recommended pharmacological and non-pharmacological therapies, and be mindful of self-care with regards to levels of physical activity,^(^ ^8^ ^)^ dietary habits, smoking,^(^ ^8^ ^,^ ^9^ ^)^ alcohol consumption,^(^ ^9^ ^)^ and prevention of complications from DM.^(^ ^9^ ^)^ Moreover, adherence to pharmacological therapy must be seen as indispensable to control DM and ensure a successful treatment.^(^ ^10^ ^)^

Non-adherence to drug therapy for DM is among the major problems faced by specialists. It also increases costs for healthcare systems due to the low rate of DM control, which leads to high morbidity and mortality rates from DM.^(^ ^11^ ^)^ The global treatment cost for the healthcare system in European countries is, on average, 1.5-fold higher than the *per capita* cost of care in relation to the general population. Moreover, these costs increase by 2 to 3.5-fold for patients who do not properly follow the drug therapy, and thus develop avoidable micro- and macrovascular complications.^(^ ^11^ ^)^ Adherence to medical treatment is the degree of agreement between medical advice and patient behavior, and is considered a process in which the subjects involved are influenced by several factors that determine treatment continuity or discontinuity.^(^ ^12^ ^)^

Considering the importance of this subject, several studies^(^ ^10^ ^,^ ^11^ ^,^ ^13^ ^)^ about adherence to drug therapy have been developed. Investigations include the association between adherence to drug treatment and a few personal and/or sociodemographic characteristics, such as sex,^(^ ^13^ ^)^ age,^(^ ^14^ ^)^ marital status,^(^ ^15^ ^)^ socioeconomic status,^(^ ^13^ ^)^ knowledge and understanding of the disease,^(^ ^15^ ^)^ perception of health risks related to DM;^(^ ^15^ ^)^ and knowledge about the costs and benefits of adequate care.^(^ ^15^ ^)^ The studies also evaluate conditions that are not directly related to the patients, but that may affect adherence to drug treatment, such as difficulty to obtain the medication and/or medical care; social support; and therapeutic complexity.^(^ ^16^ ^)^ Although these investigations bring a lot of knowledge, there is still a lot of ground to cover, and they do not definitively explain the large resistance patients have to strictly follow drug treatments.

The hypotheses about factors that affect adherence to drug treatment seem to be directly related to the patient’s health literacy level, which is an emerging theme in the literature, especially due to its association to poorer health outcomes. Health literacy is about personal, cognitive and social skills that determine one’s ability to assess, understand and use the health-related information that is necessary to promote and/or maintain good health conditions.^(^ ^17^ ^)^ A low level of health literacy affects appropriate adherence to treatment because the complex pharmacological treatment for DM requires patients to understand and apply their knowledge and they are often not able to do that.^(^ ^18^ ^)^ Previous studies^(^ ^13^ ^,^ ^19^ ^)^ suggested a direct relation between health literacy and adherence to drug treatment, when relating level of education to adherence or non-adherence to treatment, and show that the lower the level of education, the higher the probability patients will give up treatment.

We need an instrument that measures health literacy levels regarding adherence to drug therapy among DM patients. Such measurements can help us find more effective strategies to ensure diabetic patients have adequate adherence to therapy and, therefore, a better control of the disease, avoiding complications and having a better quality of life.

When creating and assessing quantitative evaluation instruments, which aim to evaluate health-related events, we must consider the results of a Delphi study, an international and multidisciplinary consensus, carried out by 43 experts. It aimed to guide such instruments and analyze the methodological quality of the studies on these events. The product of this Delphi study was the COnsensus-based Standards for the selection of health Measurement Instruments (COSMIN), and the study established a set of parameters organized into four domains: reliability, validity, responsiveness, and interpretability.^(^ ^20^ ^)^ Reliability is about the quality of the study, considering internal consistency, reproducibility, and control of random and systematic errors. Internal consistency describes the presence of a correlation between the different items that compose an instrument and between each item and the total score of the scale; that is, the homogeneity of the instrument. Reproducibility is the ability to consistently reproduce a result in space and time, with the same or different observers, showing stability, homogeneity and equivalence among different observers. Measurement errors refer to random or systematic errors of a study participant’s score, which is not attributed to true changes in the construct to be measured; that is, in the health-related event that is being investigated among a study’s participants.^(^ ^20^ ^,^ ^21^ ^)^ The validity of a measurement instrument is about its ability to accurately measure the studied phenomenon. Responsiveness is about an instrument’s ability to detect changes in the construct to be measured considering time, when events that could promote said changes are observed. Lastly, interpretability is the degree to which someone could infer qualitative results and quantitative values from a construct designed from the assessment of a health-related event. It is worth noting that interpretability is not considered a measurement property.^(^ ^20^ ^,^ ^21^ ^)^

There are, in the literature, instruments that assess levels of health literacy,^(^ ^22^ ^)^ such as instruments that evaluate health literacy among individuals with DM.^(^ ^23^ ^)^ However, no records were found of one instrument that assesses the level of health literacy specifically related to adherence to drug treatment among diabetics.

## OBJECTIVE

To prepare an instrument that evaluates health literacy related to adherence to drug treatment among diabetic patient, identify validity of its content, and estimate its reliability.

## METHODS

This is a pilot study. The first stage used to create the instrument to evaluate health literacy regarding adherence to drug treatment among DM patients was a literature review, taking into account studies that assessed health literacy and adherence to drug therapy, independently and through other methods. We also used available publications about DM, its treatment and management. The instrument was named *Alfabetização em Saúde Relacionada à Adesão Medicamentosa entre Diabéticos* (ASAM-D) (Health Literacy Related to Adherence to Drug Treatment among Diabetic Patients).

After the literature review, the instrument was structured with base on the Short Assessment of Health Literacy for Portuguese-speaking Adults (SAHLPA-18) (Annex 1), which estimates the health literacy level of adults by evaluating their skills of association and comprehension of common medical terminology.^(^ ^22^ ^)^ ASAM-D included 18 words related to DM and its treatment.

During the second stage, we verified the adequacy and consistency of items included in ASAM-D through the content validation technique, which lets us identify, through the analysis of experts, if the variables established in the evaluative components can in fact make the assessment as proposed.^(^ ^24^ ^)^ The instrument was presented to and evaluated by five expert judges (two endocrinologists, two nurses, and one dental surgeon) who were invited by convenience due to their professional experience with DM patient care. The instrument content validity was tested by the Committee of Evaluators composed by the abovementioned professionals.

The steps of the validation process were: step one – we requested the participation of professionals as evaluators, and they signed the acceptance and authorization form. The judges were also told, through a short instruction text, that, at first, they were only to give their confidential, individual opinion in the instrument, and that the instrument was created with base on the searched literature and epidemiological studies that were previously used in other works. They were also informed about the objectives, methods and rationale used to design the instrument, and that they would later participate in a collective meeting with the instrument designers, in order to reach a final version.

Once the judges agreed to participate, the second step was to ask them to evaluate every word in the document, considering their properties and ability to evaluate health literacy regarding pharmacological adherence among DM patients. The judges were asked to write suggestions and comments to improve the evaluated words.^(^ ^25^ ^)^

The third and final step of the content validity process happened with the discussion group that included all the judges that evaluated the instrument, so that a final version, with all necessary adjustments, could be made.

After including all suggestions, the instrument was ready to be applied to part of the population to be studied, aiming to determine the instrument’s reliability; that is, to guarantee that the results obtained would be the same when the instrument was used at a different time, a different place, and by other people with the same purpose.^(^ ^24^ ^-^ ^26^ ^)^

In the third stage, we estimated ASAM-D’s reliability, which was determined through its application with the test/retest method on a sample of 62 diabetic patients registered in Family Health Strategy units (ESF - *Estratégia Saúde da Família* ) (Brazilian health program). The interval between the test and the retest was between 7 and 14 days. Before the interview, participants were asked to read and sign the informed consent form. We used the following inclusion criteria: being registered at ESF centers, aged over 18 years, and, in the case of elderly patients, having reached the minimum required score in the Mini-Mental State Examination.

After the application of the test/retest, the database was consolidated, and statistical analyses were performed using the software Statistical Package for the Social Sciences (SPSS), version 20.0 for Windows and Excel. The evaluation of the internal consistency of items that compose the instrument was carried out through Cronbach’s alpha. The following thresholds were used as reference: alpha <0.30 (very low); alpha between 0.30 and 0.60 (low); alpha between 0.60 and 0.75 (moderate); alpha between 0.75 and 0.90 (high); alpha >0.90 (very high).^(^ ^27^ ^)^ The instrument’s reliability was measured through the calculation of the agreement by estimating Kappa coefficients. The following criteria of Kappa value interpretation were considered: no agreement (<0); poor agreement (0 to 0.19); reasonable agreement (0.20 to 0.39); moderate agreement (0.40 to 0.59); substantial agreement (0.60 to 0.79); and excellent agreement (0.80 to 1.00).^(^ ^28^ ^)^

This research project was approved by the Research Ethics Committee of *Universidade Estadual de Montes Claros* (UNIMONTES), under no. 764.743/2014, CAAE: 34687414.0.0000.5146.

## RESULTS

Of the 62 study participants, 83.9% were female. Mean age was 54.9 years (SD=9.97), minimum age was 29 and maximum was 77 years. Level of education varied between 0 and 12 years or more of schooling (X=5.63; SD=3.99), and 40.3% of individuals studied between 1 and 4 years in total. Among the participants, 59.7% were aged between 40 and 59 years. The most frequent occupation was homemaker, as shown in table 1 .

**Table 1:** Sociodemographic data of patients registered in the Family Health Strategy program

Variables	n (%)
Sex	
Male	10 (16.1)
Female	52 (83.9)
Stratified age, years	
20-39	4 (6.5)
40-59	37 (59.7)
60-79	21 (33.9)
Level of education, full years of schooling	
0	5 (8.1)
1-4	25 (40.3)
5-8	19 (30.6)
9-11	10 (16.1)
≥12	3 (4.8)
Occupation	
Homemaker	42 (67.7)
Retired	5 (8.1)
Recyclable waste picker	1 (1.6)
Hairdresser	1 (1.6)
Teacher	2 (3.2)
Retailer	1 (1.6)
Rural worker	1 (1.6)
Brick-layer	3 (4.8)
Small business owner	1 (1.6)
Seamstress	1 (1.6)
Lens surfacing technician	1 (1.6)
Merchant	2 (3.2)

Table 2 shows the results of the content validation process, with the variables and corresponding items that made up the initial and the final ASAM-D versions. The first version was assessed by the evaluators so it could be improved, and thus a final version of the instrument was reached. A total of 18 cards were made and presented to the participants. The final version of ASAM-D shows the correct association in bold, in order to make it easier for the interviewer to see and consolidate the results.

**Table 2:** Main alterations between the first and the last version of the health literacy assessment instrument related to adherence to drug treatment among DM patients (ASAM-D)

Item	First version (words)		Last version (suggestions)
1	Insulin – Injectable/oral		Insulin – injection/food
2	Hyperglycemia – high glucose/sweating		High glucose – Hyperglycemia/sweating
3	Tablet – measurement/oral		Tablet – length/oral
4	Diabetes – salt/disease		Diabetes – pressure/disease
5	Glycemia – hypertension/test		Glycemia – hypertension/test
6	Hypoglycemia – malaise /iron		Hypoglycemia – malaise/anemia
7	Per oris – muscle/mouth		Per oris – mouth/leg
8	Medication – control/candy		Medication – tablet/candy
9	Glucose – salt/sugar		Glucose – flour/sugar
10	Dose – quantity/sweet		Dose – quantity/validity
11	Injectable – subcutaneous/plaster		Injectable – subcutaneous/foot
12	Package insert – cake/orientation		Package insert – advertising/orientation
13	Prescription – dessert/medical		Prescription – salt/medical prescription
14	Treatment – control/cure		Treatment – control/cure
15	Decompensated – expensive/altered		Decompensated – expensive/uncontrolled
16	Continuous use – uninterrupted/large		Continuous use – uninterrupted/long
17	Prescription – Medical/discretion		Prescription – Medical/discretion
18	Side-effect – lateral/unwanted		Side-effect – lateral/unwanted

**Final version of ASAM-D**			

**Main word**	**Association word**		**Points**

1. Insulin	( ) **Injection**	( ) Food	( ) I don’t know
2. High glucose	( ) **Hyperglycemia**	( ) Sweating	( ) I don’t know
3. Tablet	( ) **Length**	( ) Oral	( ) I don’t know
4. Diabetes	( ) **Pressure**	( ) Disease	( ) I don’t know
5. Glycemia	( ) **Hypertension**	( ) Test	( ) I don’t know
6. Hypoglycemia	( ) **Malaise**	( ) Anemia	( ) I don’t know
7. *Per oris*	( ) **Mouth**	( ) Leg	( ) I don’t know
8. Medication	( ) **Tablet**	( ) Candy	( ) I don’t know
9. Glucose	( ) **Flour**	( ) Sugar	( ) I don’t know
10. Dose	( ) **Quantity**	( ) Validity	( ) I don’t know
11. Injectable	( ) **Subcutaneous**	( ) Foot	( ) I don’t know
12. Package insert	( ) **Advertising**	( ) Orientation	( ) I don’t know
13. Prescription	( ) **Salt**	( ) Medical prescription	( ) I don’t know
14. Treatment	( ) **Control**	( ) Cure	( ) I don’t know
15. Decompensated	( ) **Expensive**	( ) Uncontrolled	( ) I don’t know
16. Continuous use	( ) **Uninterrupted**	( ) Long	( ) I don’t know
17. Prescription	( ) **Medical**	( ) Discretion	( ) I don’t know
18. Side-effect	( ) **Lateral**	( ) Unwanted	( ) I don’t know

**Example of the cards shown to participants**			

Insuline			
Injection			Food

At the end of the content validation stage, the psychometric properties of the measurement instrument were ready to be evaluated. For the internal consistency of ASAM-D, results showed a total Cronbach’s alpha of 0.77 on the test/retest, which demonstrated elevated consistency among all items of the scale that assessed the health literacy of DM patients in relation to adherence to drug therapy.

As shown in table 3 , the instrument’s reliability showed a variation of Kappa values between 0.31 and 1.00 for the items included in ASAM-D, thus demonstrating reasonable to excellent agreement for items that assess the health literacy of diabetic patients in relation to pharmacological adherence. The lowest Kappa value was 0.31 and was found in the item “high glucose – hyperglycemia/sweating”, which shows reasonable agreement. The highest Kappa value was 1.00, found in 5 of the 18 items of the scale, showing excellent agreement. The rest of the items showed agreement that varied between substantial and excellent.

**Table 3:** Reproducibility estimation (simple Kappa coefficient) of the assessment instrument health literacy related to adherence to drug treatment among diabetic patients (ASAM-D)

Main word	Association words		Kappa
1. Insulin	Injection	Food	0.77
2. High glucose	Hyperglycemia	Sweating	0.31
3. Tablet	Length	Oral	0.81
4. Diabetes	Pressure	Disease	0.70
5. Glycemia	Hypertension	Test	0.96
6. Hypoglycemia	Malaise	Anemia	0.95
7. *Per oris*	Mouth	Leg	0.91
8. Medication	Tablet	Candy	1.00
9. Glucose	Flour	Sugar	1.00
10. Dose	Quantity	Validity	0.93
11. Injectable	Subcutaneous	Foot	0.87
12. Insert	Advertising	Orientation	1.00
13. Prescription	Salt	Medical prescription	1.00
14. Treatment	Control	Cure	1.00
15. Decompensated	Expensive	Uncontrolled	0.79
16. Continuous use	Uninterrupted	Long	0.93
17. Prescription	Medical	Discretion	0.95
18. Side-effect	Lateral	Unwanted	1.00

## DISCUSSION

When constructing a new measurement instrument about health-related events, it is necessary to define the domains, items and response scales based on behavior, objectiveness, simplicity, clarity, precision, validity, relevance, and interpretability criteria. When ASAM-D was created, all these factors were taken into account to remove any item that was ambiguous, incomprehensible, vague, that included double questions, jargons and/or that might suggest opinions or values.^(^ ^20^ ^,^ ^23^ ^,^ ^25^ ^)^

When a researchers create a measurement instrument, they are always aware that it is not always possible to guarantee that every domain/item involved in the researched theme will be contemplated, but the attempt is to always address the most representative aspects of each studied dimension or subject – that is why it is crucial to submit the instrument to a group of expert judges, as was done in this case. The evaluators have the task of checking the instrument’s validity through content verification and by checking if the tool is on par with the reality to be researched and with the target audience for whom it was developed.^(^ ^25^ ^)^

The literature states that new research instruments, before being applied to their target population, should be evaluated for their psychometric properties. In this study, they were tested through a reliability calculation. To determine ASAM-D’s reliability, we followed the parameters recommended by the international literature, organized and established according to the domains of the checklist COSMIN, and the adjustability of this quantitative evaluation instrument was tested through the estimations of its reliability: internal consistency and reproducibility.^(^ ^20^ ^,^ ^21^ ^)^

The results related to internal consistency of ASAM-D showed a Cronbach’s alpha of 0.773, which demonstrates the stability and consistency of the instrument proposed by this study.^(^ ^21^ ^,^ ^25^ ^)^ The reproducibility, reliability or the test/retest method estimate the total variance, which is the outcome of “true” differences between the participant’s answers and/or of results from interviews conducted at two different moments. The instrument ASAM-D presented satisfactory reproducibility because the results found for most items included in the scale show excellent agreement, without disagreement between the data collections and interviews, and that the items used in the scale to evaluate health literacy among DM patients, regarding adherence to treatment, are stable and consistent, as shown by the Kappa values. This proves that the reproducibility here presented guarantees that temporal variations in the indicators show real variations in the behavior of the population, and not an instability of the indicators.^(^ ^25^ ^)^

The analyses of the results related to the 18 items included in the instrument ASAM-D show that the lowest Kappa value found was 0.31 for the item “high glucose – hyperglycemia/sweating”. Although this value suggests reasonable agreement, it may show that the item includes concepts that are not understood by the participants, considering all other items showed Kappa values between 0.70 and 1.00, demonstrating substantial to excellent agreement. Using this instrument to assess health literacy levels can help us provide assistance to DM patients. The meaning of the word “hyperglycemia” was clear among the participants of this study; however, if a DM patient does not know what hyperglycemia means, that is a cause for concern considering patients adherence to a healthy life style can be improved if they understand their own health condition.

Regarding the reproducibility of the instrument ASAM-D, it is noteworthy that, of the 18 items included in the scale, 13 obtained Kappa values above 0.80, showing excellent agreement.

This study has a few limitations. We evaluated the instrument’s psychometric properties (reliability and internal consistency), and it is important to highlight that they are not static, that is, they may vary depending on the target population of the study, which, in this case, consisted of patients from only two primary care units. Considering the study was conducted in only one context about only one disease, this may limit the generalization of the results. Cronbach’s alpha analyses were not done considering the relation from item to item, which compromises result interpretation because it was not possible to evaluate precision by verifying if the items were redundant or insufficient. Moreover, we did not conduct a construct analysis (factorial analysis) because the number of interviewees was not enough for that. Other investigations can identify possible dimensions of the construct.

On the other hand, the use of this instrument in longitudinal studies can identify its responsiveness and contribute to optimize and establish priorities regarding a better adherence to drug treatment among DM patients, and work as a source to build health indicators and health maintenance tools.

## CONCLUSION

The items in the instrument are clear and adequate to reach the proposed objectives, which means the choice of theory was appropriate and the results of the content validation process suggest that all necessary changes to the adopted model were made. The evaluation instrument for health literacy related to adherence to drug therapy among diabetics patients was considered reliable because it showed stability; that is, acceptable internal consistency and reproducibility. However, when using the instrument, one must consider the measurement errors it might produce.

## Annex 1

**Table:**

Short Assessment of Health Literacy for Portuguese-Speaking Adults (SAHLPA-18)* Instructions for the examiner				
SAHLPA-18 evaluates a patient’s ability to pronounce and understand common medical terminology. The test can be used by healthcare professionals or researchers to estimate health literacy levels among adults. The test must be administered with printed cards that contain the medical term in bold and the two association words underneath.				

**Instructions for the examiner:**				

Before beginning, have the flash cards and the score sheet to write down the answers.				
Say:				
«I will now show you a few cards with three words. First, I would like you to read the word at the top out loud. Then, I will read the two bottom words and I would like you to tell me which of them is more related to the word at the top. If you do not know the answer, say “I don’t know” – don’t try to guess.»				
Show the first card.				
Say:				
«Now, please read the word at the top out loud.»				
Then, read the two association words and say:				
«Which of these words is more related to the word at the top. If you do not know the answer, say “I don’t know”.»				
Repeat the instructions for the following items until the patient is comfortable with the process.				
The item is considered correct only when the patient has both the pronunciation and the association right. Each correct item is worth 1 point and the total score is obtained by adding all the items, varying between 0 and 18.				
A score between 0 and 14 suggests inadequate health literacy.				

**Main word**	**Association words**			

1. Osteoporosis	( ) **Bone**	( ) Muscle	( ) I don’t know	
2. Pap smear	( ) **Test**	( ) Vaccine	( ) I don’t know	
3. Miscarriage	( ) Marriage	( ) **Loss**	( ) I don’t know	
4. Hemorrhoids	( ) **Veins**	( ) Heart	( ) I don’t know	
5. Abnormal	( ) Similar	( ) **Different**	( ) I don’t know	
6. Menstrual	( ) **Monthly**	( ) Daily	( ) I don’t know	
7. Behavior	( ) Thought	( ) **Conduct**	( ) I don’t know	
8. Seizure	( ) **Dizzy**	( ) Calm	( ) I don’t know	
9. Rectal	( ) **Watering can**	( ) **Suppository**	( ) I don’t know	
10. Appendix	( ) Itchiness	( ) **Pain**	( ) I don’t know	
11. Arthritis	( ) Stomach	( ) **Joints**	( ) I don’t know	
12. Caffeine	( ) **Energy**	( ) Water	( ) I don’t know	
13. Colitis	( ) **Intestine**	( ) Bladder	( ) I don’t know	
14. Gallbladder	( ) Artery	( ) **Organ**	( ) I don’t know	
15. Jaundice	( ) **Yellow**	( ) White	( ) I don’t know	
16. Prostate	( ) Circulation	( ) **Gland**	( ) I don’t know	
17. Incest	( ) **Family**	( ) Neighbors	( ) I don’t know	
18. Testicle	( ) Egg	( ) **Sperm**	( ) I don’t know	

* Original instrument translated into Portuguese granted by the author.^(22)^
